# Yield of Smear Microscopy and Radiological Findings of Male and Female Patients with Tuberculosis in Abuja, Nigeria

**DOI:** 10.1155/2010/241659

**Published:** 2010-07-20

**Authors:** Lovett Lawson, Mohammed A. Yassin, Alex N. Onuoha, Andrew Ramsay, Rachel R. M. Anderson de Cuevas, Sally Theobald, Peter D. O. Davies, Luis E. Cuevas

**Affiliations:** ^1^Zankli Medical Centre, Plot 1021, B5 Shehu Yar'adua Way, P.O. Box 7745, Abuja, Nigeria; ^2^Liverpool School of Tropical Medicine, Pembroke Place, Liverpool L3 5QA, UK; ^3^Faculty of Medicine, University of Liverpool, Liverpool L693Bx, UK

## Abstract

*Objective*. To describe the yield of smear-microscopy and radiological findings by male and female patients with symptoms of tuberculosis in Abuja, Nigeria. *Methods*. Patients ≥15 years old with cough for >3 weeks submitted 3 sputum samples for smear microscopy. One specimen was cultured using MGIT-960. All patients had lung X-rays and screened for HIV. *Results*. were more likely to be smear-positive than females (262/774 [34%] and 137/547 [25%], *P* < .01), but similar proportions of males and females were culture-positive (437/691 [63%] and 294/495 [59%], *P* = .09). 317/626 (50.6%) males and 249/419 (59.4%) females were HIV-positive (*P* < .005). Among culture-positives patients, HIV-infected males were less likely to have positive smears than HIV-negative males (49.2% versus 66%, *P* = .001). Among females, smear positivity did not vary with HIV (46.4% for HIV-positive and 52.9% for HIV-negative, *P* = .38). Of 274 culture-confirmed TB cases, 226 (82.5%) had cavities, and 271 (99%) had ≥1 lung areas affected. HIV-positive males were more likely to have lung cavities than HIV-positive females (85% versus 69%, *P* < .04) and to have ≥3 lung areas affected (*P* = .03). *Conclusion*. Differences in the yield of smear-microscopy, culture and X-rays on presentation are due to several factors including HIV coinfection and gender.

## 1. Introduction

Tuberculosis (TB) remains a major public health problem causing about 1.3 million deaths each year [1]. There is some evidence that men have a higher risk of developing active TB than women [2, 3], and more symptomatic men attend health services and undergo sputum examinations than women [4]. Women are also less likely to produce good quality specimens and high numbers of acid fast bacilli (AFB) in sputum samples [5]. The clinical presentation also varies between men and women, with the latter being less likely to have haemoptysis [6], and men often having more extended lung involvement at the time of diagnosis [7]. The advent of the human immunodeficiency virus (HIV) has made these interactions even more complex, as HIV has sex and age specific patterns of infection, and the virus overrides immune response differences associated with sex. At the same time, women can have less accessibility to services and a different perception of the disease than men [8], resulting in different service utilisation and a higher number of males being diagnosed with Pulmonary TB (PTB) than females. The causes of gender disparities in TB are thus likely to be a combination of biological and socially-constructed gender patterns [9, 10] which are further confounded by HIV. 

Despite worldwide description of these differences, there is limited information on whether the smear microscopy and clinical presentation disparities also occur in West Africa and how they alter with HIV coinfection. This study therefore describes the yield of smear microscopy and X-ray findings for male and female patients with chronic cough attending health services in Abuja, Nigeria and how these parameters varied in the presence of HIV coinfection.

## 2. Materials and Methods

This was a cross-sectional study of patients ≥15 years old attending eight district hospitals in Abuja's Federal Capital Territory of Nigeria with cough of >3 weeks duration. Consecutive patients were enrolled prospectively from September 2003 to December 2004. All patients were asked to submit three sputum samples for examination, following the routine screening procedures for patients with chronic cough suspected of having PTB. One sample was collected on the spot at the time of first consultation; a second sample was collected the following morning; a third sample was obtained at the time where the patients brought the morning samples to the laboratory (second on the spot). Smears prepared from these specimens were stained using the standard hot Ziehl-Neelsen method, examined using direct smear microscopy and graded according to the International Union against TB and Lung diseases (IUATLD) scale [11]. The best quality specimen of the three was cultured using an MGIT 960 system (Becton Dickinson International, Distribution Centre, Laagstraat 57, 9140, Temse, Belgium) at Zankli Medical Centre in Abuja, Nigeria. All patients with smear-positive microscopy were routinely offered HIV counselling and testing, while patients with smear negative microscopy were offered HIV counselling and testing if the hospital staff considered that this was warranted for clinical management. 

Chest X-rays were obtained from all patients with positive smear microscopy to categorise their lung involvement. X-rays were categorized following a simple scheme that is easily replicated. This scheme has been validated and used for grading radiographies of patients with chronic cough and PTB [12]. Two readers graded the X-rays independently, and radiographs were scored according to the number of lung areas with abnormalities and the presence and size of cavities. Each area of the plain posteroanterior chest X-ray is therefore equated with one sixth of the whole lung fields visible. Each area was classified as either 0 (normal) or 1 (affected) with the total area score ranging from 0 to 6. Cavities were scored according to the combined diameter of all cavities in both lungs. The absence of cavities was scored 0; cavities with a total diameter <2 cm were graded 1; those with total diameter ≥2 cm and ≤4 cm were graded 2; those with >4 cm were graded 3. Discrepancies >1 in either of the scores between the readers were resolved by discussing the grades to reach a consensus. 

Patients were enrolled after obtaining informed consent. Ethical approval for the study protocol was obtained from the ethics committee of the Liverpool School of Tropical Medicine and the Gwagwalada Specialist Hospital and the Department of Health Services of the Federal Capital Territory of Abuja.

## 3. Results

**Figure 1:**
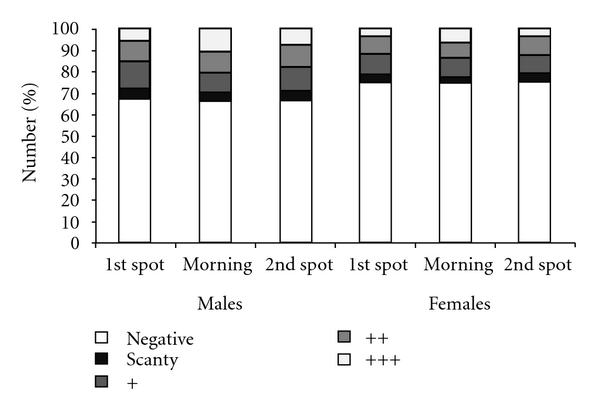
Smear grades of first spot, morning, and second spot sputum specimens by sex.

One thousand three hundred and twenty one patients, 774 (59%) males and 547 (41%) females, were enrolled. Male TB suspects were older than females (means (SD) of 35 (11) years and 31 (12) years, resp., *P* = .001) and had more prolonged cough duration (medians of 8 and 6 weeks, resp., *P* = .04). Three hundred and ninety nine (30%) patients were smear-positive (≥1 smear with >1 AFB). Morning samples had slightly higher smear grades (++ and +++) than on the spot specimens, but the proportions of positive morning and spot samples (≥1 AFB per 100 high-power fields) were similar. Males were more likely to have positive smear microscopy than females (262 (34%) and 137 (25%), resp., *P* < .001) and to have higher smear grades (++ and +++) in the morning (20%) and 2nd on-the-spot smears (17%) than females (13% and 11%, resp., *P* < .01), as shown in Figure 1. Smear positivity was associated with age. Smear-positive and smear-negative males had a mean age of 33 and 36 years compared to 28 and 34 years for smear-positive and smear-negative females (*P* = .001). A total of 1186 (90%) patients had culture results and of these, 731 (61.6%) were culture-positive. The proportion of culture-positive cases did not vary by gender, as 63% (437/691) of the males and 59% (294/495) of the females were culture-positive (*P* = .09). 

Three hundred and seventeen (41%) of 626 (81% tested) males and 249 (46%) of 419 (77% tested) females were HIV positive (0.005). Among culture-confirmed cases tested for HIV (625), HIV-infection rate increased with age for males (peak age group = 45−54 years) and decreased for females (peak age group = 25−34 years). Smear positivity rate decreased with age among both sexes, as shown in Table 1. Among males, HIV infected patients were less likely to have positive smear microscopy than HIV negatives (49.2% versus 66%, *P* = .001). Among females, smear-microscopy results did not vary by HIV status (46.4% for HIV-positive and 52.9% for HIV-negative, *P* = .38), as shown in Table 2.

**Figure 2:**
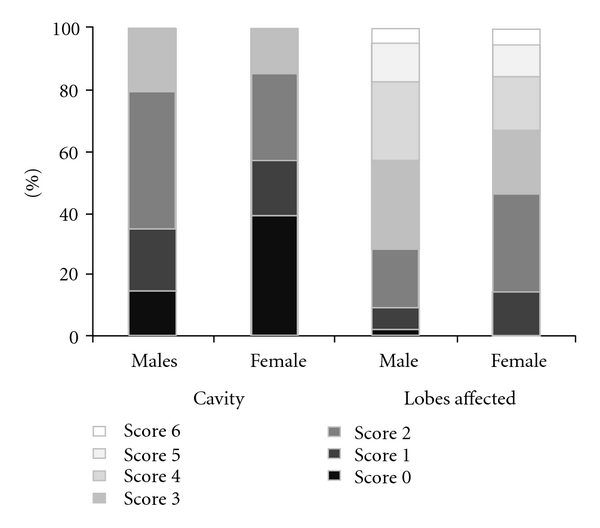
X-ray cavity and area affected scores of culture positive TB cases by gender.

X-rays were available for 274 patients with culture-confirmed PTB. Of these, 226 (82.5%) had cavities (scores ≥1) and 271 (99%) had at least one lung area affected. The X-Ray scores by HIV status and gender are described in Table 3 and Figure 2. Among HIV-positive patients, males were more likely to have lung cavities than females (85% versus 69%) (*P* < .04) and to have a higher number of affected lung areas, with 52% of the males having ≥ 3 areas affected compared with 31% of the females (*P* = .03). These gender differences disappeared for HIV-negative patients, as the same proportion of males and females had cavities (87% and 88%), and 39% of males and 38% of females had ≥3 lung areas affected.

## 4. Discussion

Sex is a key factor modulating innate immunity, host response to infection, and disease progression. Infections from parasites, viruses, and bacteria are more frequent and severe in males than females [13], and women with severe trauma are less likely to experience infections such as pneumonia, sepsis, and multiple organ trauma than men [14, 15]. Sex hormones influence energy, glucose, and lipid metabolism, and sex steroids regulate inflammatory cytokines that cause the hypermetabolism and catabolism associated with acute phase responses [16]. Gender (the socially constructed roles, behaviours, activities, and attributes that a given society considers appropriate for men and women) is also a key factor modifying health-seeking behaviour, determining accessibility to services, disease perception, and confidence in the health services [17, 18].

The high proportion of patients with positive cultures (61.6%) in this case series could be related to the recent introduction of the DOTS programme in this setting, which was initiated during the study period. It is thus likely that patients with chronic conditions who did not have access to diagnosis would be given priority for screening by the attending physician, and these patients would be more likely to be culture-positive.

The case series presented here confirms that males and females with chronic cough have different clinical characteristics at the time of presentation to the health services. The data however does not support a distinction between the differing effects of gender and sex, which would be useful to identify strategies to address how gender roles and relations shape vulnerability to TB and access to services.

In most developing countries, more males attend the health services than females [19], and more men have positive smear microscopy than women [5, 17, 20]. This discrepancy was reduced by training patients how to produce good quality sputum in Pakistan, suggesting that it is possible to change the yield by modifying behaviour [5], or using new microscopy methods. Very few studies however have described whether the higher yield of smear microscopy also results in a higher proportion of cultures being positive. In this study, similar proportions of males and females had positive cultures. Among culture-confirmed TB cases, males were more likely to be smear-positive than females and HIV-negative patients were more likely to be smear-positive than HIV-positive patients. The decrease in sensitivity of smear microscopy was observed in both sexes. 

Males were more likely to have cavitations and more extensive lung involvement than women. In Vietnam, men were more likely to have more advanced radiological findings than women, despite a similar duration of time from symptom onset to diagnosis [7]. Although cavities are not formed in all patients with caseous lesions [21], the walling-off of lesions and the creation of cavities are important to the continuous transmission of the bacilli and cell-mediated responses to bacillary components, the presence of caseous necrosis and delayed-type hypersensitivity (DTH) is necessary for the formation of cavities. Radiological findings may thus be related to sex-specific differences, although the intricate pathogenesis of cavity formation has only recently become a more prominent aspect of research [21]. 

HIV-infected TB patients have impaired DTH responses due to a deficiency in CD4 T cells and are less likely to develop cavitary disease [22]. Cavitations and lung involvement were more pronounced among HIV-uninfected males than females, while among HIV-infected patients, both genders had similar lung involvement. Coinfected females however were less likely to have cavitations than males, as previously reported [20]. These differences may be confounded by the epidemiological characteristics of HIV-infection. For example, men tend to acquire HIV infections at a later age and are more likely to smoke and drink alcohol than women. Age, smoking, and drinking have been associated with immunosuppresion and increased propensity to develop progressive forms of TB [17]. 

Smear microscopy and radiological differences between male and female patients with symptoms of TB are thus likely to reflect a complex interaction of social and biological differences [17]. Further studies are necessary to elucidate the immunological mechanisms at play and to consider how these could be harnessed to develop preventive and curative approaches that are sex/gender specific. Health systems need to consider the consequences of both sex and HIV per se on the clinical presentation and prognosis of TB and the effect those differences in societal gender roles have on accessibility to services.

## Figures and Tables

**Table 1:** Age and gender distribution of culture positive cases by their HIV and smear microscopy status.

Age (yrs)	Male				Female			
	HIV+	HIV−	SM+	SM−	HIV+	HIV−	SM+	SM−
15–24	16 (26.7%)	44 (73.3%)	35 (55.6%)	28 (44.4%)	40 (59.7%)	27 (40.3%)	42 (56.8%)	32 (43.2%)
25–34	81 (49.7%)	82 (50.3%)	119 (64.3%)	66 (35.7%)	72 (62.6%)	43 (37.4%)	58 (42.3%)	79 (57.7%)
35–44	57 (59.4%)	39 (40.6%)	46 (40.4%)	68 (59.6%)	21 (58.3%)	15 (41.7%)	16 (34.8)	30 (65.2%)
45–54	26 (60.5%)	17 (39.5%)	22 (43.1%)	29 (56.9%)	6 (40%)	9 (40%)	5 (23.8%)	16 (76.2%)
55–64	6 (42.9%)	8 (57.1%)	4(23.5%)	13 (76.5%)	0 (0)	5 (100%)	2 (20%)	8 (80%)
65–74	3 (60%)	2 (40%)	1 (20%)	4 (80%)	1 (33.3%)	2 (66.7%)	1 (33.3%)	2 (66.7%)
75–84	0 (0)	1 (100%)	1 (100%)	0 (0)	0 (0)	1 (100%)	0 (0)	2 (100%)
85 and above	(0)	1 (100%)	1 (100%)	0(0)	0 (0)	0 (0)	0 (0)	1 (100%)
Total	189 (49.3%)	194 (50.7%)	229 (52.4%)	208 (47.6%)	140 (57.9%)	102 (42.1%)	124 (42.2%)	170 (57.8%)

SM = smear-microscopy.

**Table 2:** Smear-microscopy results by HIV status and gender in culture-positive patients.

HIV	Male		Female		
	Positive	Negative	Positive	Negative	All
	*N* = 189 (49.3%*)	*N* = 194 (50.7%)	*N* = 140 (57.9%*)	*N* = 102 (42.1%)	*N* = 625
Smear-positive	93 (49.2%**)	128 (66%)	65 (46.4%**)	54 (52.9%)	340 (54.4%)
Smear-negative	96 (50.8%)	66 (34%)	75 (53.6%)	48 (47.1%)	285 (44.6%)

*HIV positivity by gender (or, 95%CI for female compared to male = 1.01−1.97, *P* = .046),

**Smear positivity among HIV-positives versus HIV-negatives, *P* < .001 for males, and *P* = .38 for females.

**Table 3:** Radiological scores of patients with positive culture by gender and HIV status.

	Cavities				Areas affected			
Score	HIV-positive		HIV-negative		HIV-positive		HIV-negative	
	Male	Female	Male	Female	Male	Female	Male	Female
	*N* (%)	*N* (%)	*N* (%)	*N* (%)	*N* (%)	*N* (%)	*N* (%)	*N* (%)
0	11 (15)	16 (31)	14 (13)	5 (12)	3 (4)	0 (0)	0 (0)	0 (0)
1	14 (19)	13 (25)	22 (21)	8 (19)	7 (10)	7 (14)	6 (6)	7 (16)
2	31 (43)	17 (33)	49 (46)	17 (40)	11 (15)	18 (35)	22 (21)	11 (26)
3	16 (22)	6 (12)	21 (20)	12 (29)	15 (21)	11 (21)	37 (35)	9 (21)
4					19 (26)	11 (21)	28 (26)	6 (14)
5					13 (18)	5 (10)	9 (9)	5 (12)
6					5 (7)	0 (0)	4 (4)	5 (12)

Cavity scores were not available for two patients.
